# Barriers and Facilitators to Patient Utilization of Non-Communicable Disease Services in Primary Healthcare Facilities in Nepal: A Qualitative Study

**DOI:** 10.21203/rs.3.rs-5324989/v1

**Published:** 2024-11-15

**Authors:** Sushmita Mali, Elizabeth C. Rhodes, Chandani Singh Nakarmi, Soniya Shrestha, Aarati Dhakal, Alina Bharati, Anupama Bishwokarma, Asmita Adhikari, Bikram Poudel, Binuka Kulung Rai, Sangita Manandhar, Surakshya KC, Dinesh Timalsena, Sashi Silwal, Meghnath Dhimal, Phanindra Prasad Baral, Felix Teufel, Sanju Bhattarai, Donna Spiegelman, Archana Shrestha

**Affiliations:** Dhulikhel Hospital-Kathmandu University Hospital; Emory University; Dhulikhel Hospital-Kathmandu University Hospital; Dhulikhel Hospital-Kathmandu University Hospital; Dhulikhel Hospital-Kathmandu University Hospital; Dhulikhel Hospital-Kathmandu University Hospital; Dhulikhel Hospital-Kathmandu University Hospital; Dhulikhel Hospital-Kathmandu University Hospital; Dhulikhel Hospital-Kathmandu University Hospital; Dhulikhel Hospital-Kathmandu University Hospital; Dhulikhel Hospital-Kathmandu University Hospital; Dhulikhel Hospital-Kathmandu University Hospital; Dhulikhel Hospital-Kathmandu University Hospital; Nepal Health Research Council; Nepal Health Research Council; Ministry of Health and Population; Emory University; Norwegian University of Science and Technology; Yale School of Public Health; Kathmandu University School of Medical Sciences

**Keywords:** Package of Essential Non-Communicable Diseases (PEN), Non-communicable diseases, Patient’s perspective, Health Belief Model (HBM), Perceived Barriers, Perceived Facilitators

## Abstract

**Background:**

The Nepalese government endorsed and implemented the Package of Essential Non-Communicable Disease Interventions (PEN) by the World Health Organization (WHO) to prevent and manage four major non-communicable diseases (NCDs): cardiovascular disease (CVD), diabetes, cancers, and chronic respiratory diseases. This study explored barriers and facilitators to patient utilization of NCD services at primary healthcare facilities in Nepal.

**Methodology:**

We conducted a qualitative study with a 35 purposive sample of patients living with one or more NCDs (hypertension, diabetes, chronic obstructive pulmonary disease (COPD/ asthma) who sought healthcare at primary healthcare facilities in 14 randomly selected districts in seven provinces in Nepal that implemented PEN. Trained qualitative researchers conducted in-depth interviews in-person in a private setting using a semi-structured interview guide developed based on the Health Belief Model in the local language. The interviews were audio-recorded, transcribed verbatim, coded inductively and deductively, and analyzed by a framework approach using Dedoose software.

**Results:**

From the perspectives of patients, key facilitators of service utilization encompassed free medicines, low-cost services, geographical and financial accessibility, less waiting time, positive interactions with health service providers, experiencing improvements in their health conditions, and support from family and peers. Barriers to utilizing services included inadequate health services (e.g., lack of medications and equipment), inaccessibility and affordability, inadequate health-related information from health service providers, low knowledge of NCD care, and lack of reminders or follow ups.

**Conclusion:**

Enhancing NCD service utilization is potentially attainable through interventions that address patients’ knowledge, self-motivation, and misconceptions. Furthermore, strengthening the availability and accessibility of crucial services such as laboratory investigations, medications, equipment, and the patient-provider relationship is crucial for sustainable implementation of PEN.

## BACKGROUND

Globally, noncommunicable diseases (NCDs) account for 74% of all deaths(1), with anticipated economic losses of 75% of the global Gross Domestic Product (GDP) by 2030(2), significantly straining healthcare systems(3). A substantial 77% of NCD-related deaths occur in low- and middle-income countries (LMICs)(3). Notably, South Asia bears a substantial NCD burden(4), with a high mortality rate of 1.9 million deaths. 39% of poor-quality service access deaths are attributable to substandard healthcare, while 36% is due to healthcare non-utilization(5). In Nepal, the year 2018 saw cardiovascular diseases (CVDs), chronic respiratory diseases (COPD), cancer, and diabetes mellitus (DM) contribute to 66% of all deaths, constituting 39% of the overall disease burden(6). In response to these alarming data, the World Health Organization (WHO) has proposed a cost-effective package of essential noncommunicable disease (PEN) Interventions.

While some factors influencing patient utilization have been identified, there remains a dearth of understanding regarding the complex interplay of socio-cultural, economic, and individual determinants affecting healthcare-seeking behaviors in the context of NCDs. Furthermore, limited data exists on the effectiveness of interventions aimed at improving NCD service utilization among patients. Patient utilization matters profoundly as it directly impacts the timely detection, management, and prevention of NCDs, thus influencing health outcomes and overall healthcare system efficiency(7). Yet, factors influencing patient utilization of services are underexplored. Existing research on NCD service utilization in Nepal has primarily centered on the viewpoint of health service providers. These studies highlight issues related to the screening, diagnosis, treatment, and follow-up of NCD patients. They also emphasize the need for a consistent supply of essential medicines and improvements in service delivery to implement the PEN interventions in Nepal(8, 9). However, evidence is needed regarding the perspectives, experiences, and satisfaction levels of patients receiving NCD care in resource-constrained settings(10). Given this gap, a comprehensive understanding of how patients perceive and engage with NCD services is crucial to refine their NCD care models. By addressing the unmet needs of patients and fostering greater patient involvement in their care, these models can contribute to improved outcomes and a higher quality of care(10–12). Therefore, a patient-centered perspective on NCD services utilization holds the potential to drive meaningful advancements in LMIC healthcare systems.

The objective of this study was to explore the factors influencing patient utilization of NCD services within primary care health facilities that have adopted the PEN interventions from the perspectives of NCD patients. The insights gleaned from our investigation will play a pivotal role in formulating demand-side strategies.

## METHODOLOGY

### Study Design and Setting

We conducted a qualitative study in Nepal, a low and middle-income country in South Asia(13). In 2015, the nation’s health system has been restructured into three tiers: the federal level consisting of seven provinces and 753 local governments. These sub-national entities possess greater autonomy and resources in the planning and managing of health services(14). Primary health facilities, overseen by local governments, offer direct services to local communities and manage health service delivery, primarily relying on mid-level health workers. Only a few government and private tertiary-level facilities in major cities care for NCD patients. Nepal’s PEN Implementation Plan (2016–2020) was developed to align with the Multisectoral Action Plan for the Prevention and Control of NCDs (2014–2020)(15). The WHO PEN intervention was adopted by Nepal, with the Epidemiology and Disease Control Division (EDCD) under the Department of Health Services being the primary agency responsible for its implementation. To enhance accessibility for Universal Health Coverage (UHC), PEN was initially introduced as a pilot project in two districts (Illam and Kailali) in 2017. Subsequently, during 2018/19, PEN’s scope was extended to cover 31 districts out of the 77 in Nepal(16). The PEN program consists of evidence-based cost-effective interventions for the prevention and management of hypertension, diabetes, asthma, chronic obstructive pulmonary disease (COPD), and breast and cervical cancer(17). The national PEN program aims to improve NCD management through early detection in primary healthcare facilities, i.e., health posts and Primary Health Care Centers (PHCCs) at the primary care level.

#### Study site and participants

We conducted the study in 14 randomly selected districts where the Package of Essential Noncommunicable (PEN) Disease Interventions (PEN-implemented districts) were implemented, representing all seven provinces. We randomly selected 105 primary healthcare facilities from these districts, from 3,794 Health Posts (HPs) and 189 Primary Health Care Centers (PHCCs)(18). Within these facilities, we purposively sampled 35 participants with non-communicable diseases(NCDs), defined here as hypertension, diabetes mellitus (DM), chronic obstructive pulmonary disease (COPD), and asthma, who were visiting the outpatient department during data collection. The inclusion criteria comprised participants aged 30 years or older and under the prescription of NCD medicines. We excluded those with severe mental and physical disabilities. We ensured diversity in the sample by recruiting participants with varying characteristics, including gender, level of education, and family history of disease.

This study received ethical approval from the Ethical Review Board (ERB) of Nepal Health Research Council (Registration no: 327/2020P) and Kathmandu University School of Medical Sciences (Approval no: 110/20). Interviewers obtained verbal and written informed consent from each participant, explaining the study’s purpose and the right to withdraw. Patient names, transcripts, and audio files were securely stored and accessible only to the research team to maintain confidentiality.

#### Sample size

The sample size was determined based on code saturation and meaning saturation principles(19), which indicates the point at which no new codes or themes are emerging from the data. After conducting 21 interviews, no additional insights or themes were identified. Subsequently, the achievement of saturation was determined through discussions with the research team, indicating that the analysis had reached a point of saturation.

#### Participant Recruitment

With the approval of health service providers, trained research assistants (RA) approached participants waiting for follow-up clinical consultations in the health facilities using a normal conversation– rapport-building approach. Once the participants agreed to engage in further conversation, we comprehensively explained the study’s purpose, procedures, confidentiality measures, and potential risks, and benefits to all potential participants. Those who provided written consent were subsequently enrolled in the study.

### Theoretical Model

**Figure 1:**
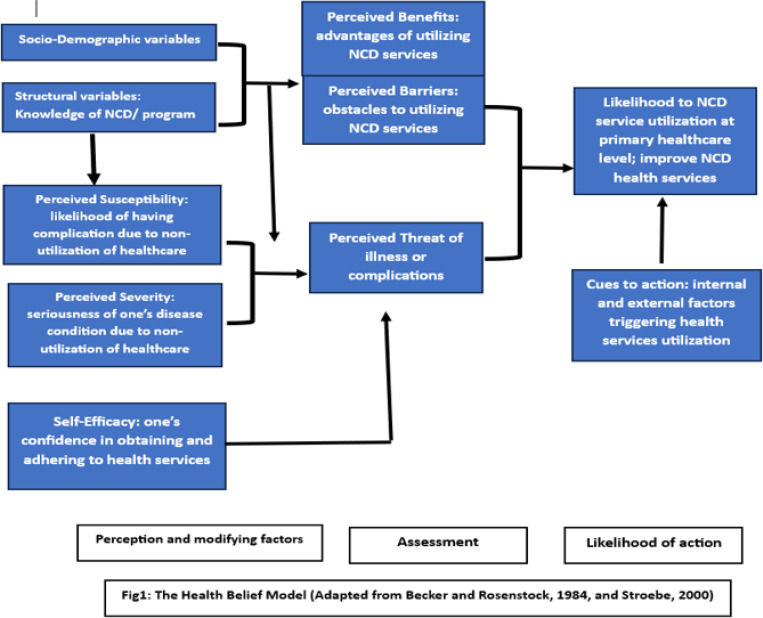
Health belief model

To guide this study, we employed the Health Belief Model (HBM), which encompasses six constructs: (1) perceived susceptibility (the perceived likelihood of having complication due to non-utilization of healthservices); (2) perceived severity (the perceived seriousness of one’s disease condition due to non-utilization of healthcare services); (3) perceived benefits (the perceived advantages of utilizing non-communicable disease services); (4) perceived barriers (the perceived obstacles to utilizing non-communicable disease services); (5) cues to action (internal and external factors that trigger health service utilization); (6) self-efficacy (the individual’s confidence in successfully obtaining and adhering to health services)(20, 21)(Fig. 1).

Applying the HBM in this study established a theoretically grounded approach to comprehending participants’ perceptions and the factors influencing their decisions to the use of health services from healthcare facilities. This understanding holds significant potential for enhancing the impact of NCD health services and tailoring future interventions to address participants’ needs and preferences effectively.

### Data collection

We conducted face-to-face in-depth interviews (IDIs) with participants, guided by a semi-structured interview guide with questions derived from the aforementioned Health Belief Model (HBM) constructs. It aimed to gather information on facilitators, barriers, and individual-level perceptions regarding NCD service utilization offered by the PEN program. The guide was finalized based on pilot test insights and team discussions.

Seven trained qualitative researchers (SM, CS, BP, AP, AA, AB, SK, BKR, SM) conducted in-depth interviews between April and October 2021. All interviewers were native Nepali speakers trained in qualitative data collection. All interviews were in Nepali, except for one that was conducted in Doteli. Interviews took place in private and quiet healthcare facility spaces. The interview duration ranged from 15 to 47 minutes, some shorter due to the patient’s limited knowledge, hesitation, or time constraints.

### Data management and analysis

We analyzed the data in six stages of framework analysis outlined by Ritchie & Spencer(22): transcription; familiarization; coding; identifying a framework; charting into matrix; and interpretation. The thematic framework analysis using HBM constructs guided the analysis. First, research assistants (RAs) transcribed all recorded interviews verbatim, and the transcripts were de-identified to ensure participant anonymity. The first author (SM) meticulously reviewed the complete transcripts while simultaneously listening to the audio recordings to verify accuracy and completeness. The data were imported into Dedoose software (version 8.03.05, later upgraded to 9.0.54). Second, the two coders (SM and CS) carefully read one-third of the transcripts to familiarize themselves with the data. They iteratively developed the codebook, organizing it into a manual containing inductive codes derived from the data, deductive codes from interview guides, and definitions and application examples. Frequent discussions occurred during the codebook development to review potential themes under each HBM construct. The research team ensured the codebook’s alignment with the HBM structure, resolving any differences under the guidance of the qualitative research expert (ER). Fourth, the codebook was used to code the first three transcripts, checking for inter-rater reliability. The coders discussed and resolved disagreements in the code application, repeating the process until they reached an 80 percent agreement. After achieving 89.6% inter-coder reliability, the coders independently applied the codes to the remaining interviews. They diligently searched, coded, and categorized excerpts to identify repetitive patterns of themes. Fifth, a spreadsheet was used to create a matrix; the data were summarized by category from each transcript and were charted into the matrix. In addition, it included interesting illustrative quotations. Finally, throughout the coding process, the coders discussed all emerging codes and meaningful patterns, providing detailed descriptions.

## RESULTS

The participants’ characteristics are provided in Table 1. Thirty-five NCD patients were interviewed, with 54% being female and ages ranging from 35 to 92 years. Among them, 63% had hypertension, and 17% had diabetes. About 49% reported a family history of NCDs. Approximately 50% of the participants had no formal education, 34% worked in agriculture, and 37% identified as homemakers.

The paper is structured to examine the facilitators and barriers influencing NCD service utilization at primary healthcare systems in Nepal, through the theoretical lens of the HBM from the participants’ perspective. By analyzing the 6 HBM constructs such as perceived susceptibility, severity, benefits, barriers, and cues to action. Further, the study sheds light on the nuanced interplay of factors influencing NCD service utilization, highlighting both the opportunities and challenges in these settings (Table 2).

### Perceived Barriers

Many health facility-related perceived barriers to NCD management emerged in the interview.

#### Health services constraints

All participants expressed frustration with the inadequate supply of medicines, lack of well-equipped infrastructure, and a shortage of skilled healthcare providers. The unavailability of essential drugs was a significant deterrent to NCD service utilization. Moreover, some participants encountered barriers in obtaining health insurance schemes, and those visiting health posts were often referred to higher centers for laboratory investigations and surgery, which added to their difficulties in accessing comprehensive care.

“The doctor is not available. Till date, we have not received anything easily… we have to buy most of the medicines as it is unavailable in the facilities… If you have bigger health issues and If the treatment is not available here, they refer to bigger health facilities, hospital.”

(DM Patient, 35 years, Male, PHCCs)

This was especially true for older participants who had difficulty managing chronic health conditions or in some cases, multiple chronic health conditions simultaneously, navigating complex healthcare services and coping with physical limitations hindering mobility and independence.

“We have been getting our medicines through an insurance scheme from Gandaki Hospital, but we are old and cannot go there. Therefore, I have come here to ask if getting the medicines from this health post is possible, but it is not available here.”

(HTN Patient, 92 years, Male, PHCCs)

Participants expressed frustration regarding the lack of access to necessary medications despite having paid for insurance. The participants highlighted encountering excuses from healthcare providers regarding medication availability or delays due to administrative procedures, such as meetings for decision-making causing delays. This experience underscores systemic challenges within the healthcare system that hinder individuals’ ability to access essential treatments promptly. Such barriers not only exacerbated the burden of chronic diseases but also contributed to feelings of disillusionment and dissatisfaction among patients who rely on insurance coverage for healthcare support.

“Because we are suffering from chronic disease, that’s why we have paid for insurance but they do not provide medicines. When ask for the medicine they make excuses of unavailablity or tell us they have to sit for meeting and decide and stuff like that.”

(DM, Female, HP)

#### Inaccessibility & affordability

Geographical accessibility posed a significant obstacle to seeking NCD care, encompassing physical distance and available transportation options. Due to these challenges, participants residing in rural hilly areas expressed that seeking healthcare elsewhere was impractical. Moreover, the region’s natural hindrances, including intense seasonal rains and landslides, further impeded participants from accessing necessary services. Individuals attending health posts recounted being directed to higher-level facilities for medication and laboratory work, resulting in added travel expenses that placed an extra financial strain. The cost associated with treatment played a pivotal role in shaping individuals’ health-seeking patterns. This often led to the difficult decision of either postponing, preceding, or discontinuing treatment, a sentiment particularly pronounced among participants from economically disadvantaged households.

“During the monsoon season, due to heavy rain, the roads are blocked. It is difficult to travel during the season. If there were a gravelled road, it would have been easy. Transportation problem and unavailability of vehicles is a big problem in our area”.

(HTN & COPD Patient, 67 years, Male, HP)

“It takes hours to reach Beni Hospital. It takes an hour for locals and 2 hours or more for those who are not from around. It takes a whole day to reach Beni, and it costs Rs. 200. That’s a lot for us: bus fare, medicine costs, lunch expenses; the trip is very costly for us.”

(DM patient, 63 years, Male, HP)

#### Limited knowledge about NCD care

The lack of awareness surrounding NCD programs, such as PEN and health insurance schemes, acted as a barrier, hindering participants from using available NCD services. Many participants acknowledged a lack of comprehensive education regarding their health conditions and the corresponding management protocols. Certain participants held reservations about relying on pharmaceutical interventions indefinitely, leading them to start medication only after exploring all conceivable alternatives, including traditional and herbal remedies. A small minority expressed concerns about potential medication side effects, which subsequently discouraged them from maintaining consistent adherence to the prescribed treatment. A prevalent lack of understanding regarding possible disease complications, combined with limited awareness of the inherent health risks and significant consequences, contributed to the delay in seeking necessary medical attention. Within this context, a subset of participants even reported instances of prematurely discontinuing medication once their presenting symptoms improved.

“I didn’t know about the drug information. If I had known that medicine for high blood pressure should be taken lifelong, I think I would not have started it.”

(DM Patient, 63 years, Male, HP)

“My hands and legs went weak. First I got treatment with a witch doctor. That did not work. I became ill, I went to Kathmandu and came to know it was high blood pressure.”

(HTN Patient, 76 years, Male, HP)

#### Inadequate health-related information from Health Service Providers

Communication breakdowns between participants and health service providers, coupled with shortcomings in delivering effective counseling, emerged as additional obstacles to the utilization of NCD services. Instances of subpar encounters where participants received insufficient information about their health conditions, exacerbated by limited doctor availability, particularly in PHCCs, were highlighted as demotivating factors. Some participants expressed dissatisfaction with the lack of counseling from healthcare providers regarding disease management, encompassing medication adherence and lifestyle adjustments. Several participants also indicated that their health service providers failed to provide insights into the various NCD-related services accessible within the healthcare facility.

“While sharing the symptoms and difficulties, nobody gave information on the actual cause of high BP or the cause of breathlessness. I don’t know what caused me HTN and Asthma… There has not been much counseling on my health conditions.”

(HTN & COPD Patient, 67 years, Male, HP)

“I have not received any suggestions from Health service providers for my conditions. Doctors do not give much time. They only ask what is the reason for our visit and write prescriptions. They do not talk much.”

(HTN & DM Patient, 53 years, Female, HP)

#### No reminders/followups

A need for follow-ups or reminders from health service providers emerged as an additional hindrance to accessing healthcare services. Participants highlighted instances of missing their scheduled follow-up visits due to conflicting priorities. Some individuals expressed that timely reminders from Health service providers could have averted these lapses in their follow-up routine.

“There has not been any contact from the Health Service providers… There’s no follow-up service. When the medicine finishes, if we do not feel well, we come ourselves for checkups. Nothing is done from the health centers.”

(DM Patient, 63 years, Male, HP)

#### Perceived Susceptibility:

Many participants lacked knowledge and understanding of the NCD healthcare services that was available in their commiunity and did not know that they require regular health-check up for NCD care. One participant expressed a low sense of vulnerability, stating, “I do not feel any danger as I have not experienced anything bad till now by not going for healthcare check-up regularly,” indicating a lack of perceived susceptibility. In contrast, another participant demonstrated a high perceived susceptibility by demonstrating motivation to NCD care utilization: “While taking hypertension medicine, I used to eat meat and spicy foods. But, after receiving counseling from my service provider, I stopped eating meat and spicy food (high sodium).”

#### Perceived Severity

Individuals’ perceptions of the severity of health conditions significantly influence their motivation to seek treatment, thus, contributing to the use of NCD services. A few participants shared that witnessing their family members’ experiences of complications for not regularly using medication or healthcare made them realize the severity of NCDs. Furthermore, seeing others suffer as a result of NCD complications like vital organ failure, disability, paralysis, or early death made them fearful of NCDs and associated morbidity and mortality due to delay in seeking healthcare. One participant who experienced two family members with NCD-related complications shared

“My brother had hypertension and did not see doctor or use medication regularly.. he died from paralysis, and I remember what he had to go through. I’m scared that I might die from paralysis, too.”

(HTN Patient, 66 years, Female, HP)

Most participants reported that their experiences of symptoms, such as dizziness, heartache, headache, breathlessness, fatigue, difficulty breathing and walking, loss of appetite, and tingly sensations, influenced their decisions to seek medical help.

“I used to have dizziness, pain in the neck, loss of appetite, body aches, and headache, so I had gone for checkups experiencing all these symptoms. Also, my brother suggested I get checked if I have DM or High blood pressure”.

(HTN Patient, 46 years, Female, HP)

#### Perceived Benefits

Several features of the health facility and family emerged that were perceived as effective in reducing complications or combatting the symptoms of NCDs.

#### Accessibility

Most participants reported that the proximity of the health facility to their homes encouraged them to use its services. Participants living nearby or with good roads to reach health facilities reported seeking services regularly, as geographical accessibility played a significant role in NCD service utilization and continuity of use. Participants highlighted the convenience and accessibility of local health services. A male diabetes patient mentioned that the proximity of the health post to his home allows him to visit easily during his free time, emphasizing the benefit of the 24-hour service availability which facilitates access during emergencies. Similarly, a 92-year-old male hypertension patient appreciated PHCCs, noting the challenges in traveling long distances and the lack of consistent transportation options, especially at odd hours. He expressed gratitude for the nearby services, which significantly enhance his ability to receive care without the need for extensive travel.

#### Short waiting time

One of the critical factors that positively influenced NCD service utilization was the reasonable and practical waiting time of under 30 minutes per visit for participants. The importance of short waiting times was a commonly reported and highly valued aspect of patient’s healthcare experiences. Several participants from both PHCCs and health posts expressed that they appreciated the relatively short waiting times for services, including consultations, health examinations, and medicine dispensing. This efficient service delivery motivated them to seek healthcare services at the health facility.

“I get the services on time, I don’t have to wait, and they respond immediately… there has not been a time when I had to return without getting checked; someone is always available.”

(HTN Patient, 92 years, Male, PHCCs)

#### Positive interaction with health service providers

The willingness of service providers to listen to participants’ concerns and exhibit positive behaviors played a significant role in making participants feel comfortable and building trust. The existence of a strong patient-provider relationship emerged as a motivating factor for participants to use services, including visiting healthcare facilities regularly and undergo screenings. Participants highly valued the information and guidance healthcare providers provided, enabling them to gain knowledge on disease management and better understand their health conditions.

“They [health service providers] are accommodating, and respond nicely. They behave politely. The doctor is excellent here.”

(HTN Patient, 40 years, Female, HP)

“Health Servive Providers behave well, provide suggestions on the do’s and don’ts; being one of the reasons to visit the health facility.”

(DM Patient, Missing age, Male, HP)

#### Services at low-cost/ free services and medicines

Many participants mentioned experiencing a positive outcome on their health condition from medication and diagnostic services they received from primary health care facilities. The satisfaction of participants with NCD services depended on several factors; free health check-ups, medicine refills facilitation, low medication costs, and low user fees for laboratory and diagnostic services. As a result of medicine consumption, many participants explicitly cited a favourable outcomes in their health condition.

“I come here to HP as they have the medicines here. Medicine is available here, it has been one year I have been taking medicine from here, previously I used to buy medicines from outside.”

(HTN & DM Patient, 52 years, Female, HP)

“The medicine from the health facility has helped to control and manage my condition..I have not visited other health facilities, taking medicines from here only

(DM & HTN Patient, age missing, Male, HP)

#### Health Insurance Scheme

The availability of free medicine, and free health check-ups or subsidized prices through social health insurance were encouraged participants to seek treatment from health facilities, particularly those from economically disadvantaged backgrounds, the financial burden associated with healthcare services served as a significant barrier to accessing necessary treatment. The provision of free medications and screenings and the option for subsidized prices through social health insurance alleviated this burden, making healthcare more accessible and affordable. Participants reported that the health insurance scheme was only accessible at PHCCs and covered free consultations, limited medicines, and laboratory tests. NCD services are limited to blood pressure screening, blood glucose tests, and medicines at health posts without social health insurance.

“Insurance has opened access to all kinds of (health) checkups, medicines, and other health-related services. The treatment cost is cheap, I tested my sugar for Rs.40 ($0.03)… My sugar was diagnosed at this facility when I came to check uric acid.”

(DM Patient, 61 years, Male, PHCCs)

#### Support from family and peers

Family and peer support were cited as significant contributors to the utilization of healthcare services. Participants described that the moral support they received from family members, friends, and neighbors was crucial in motivating them to initiate treatment and start medication for their health conditions. The various forms of support provided by family and peers included encouragement for checkups, medication reminders, assistance with travel, especially for those with mobility challenges or transportation limitations, medication refilling, financial support, and support in household chores such as cooking and cleaning. This encouragement and assistance contributed to better treatment adherence, increased health awareness, and improved health outcomes.

“My husband took me to the Nepalgunj Hospital for treatment and got me medicine. He always takes me to the health facilities for checkups…..”

(HTN Patient, age missing, Female, PHCCs)

“At the time of sickness, without my friends, I might have lost my life when my BP (blood pressure) was increased. I recall a time when my friends looked after my cattle, letting me take a rest under the shadow and seek health care.”

(HTN Patient, 61 years, Female, HP)

### Cues to action

#### Experiences with Illness

Observing family members endure severe conditions motivated participants to seek medical care proactively. Some participants noted that their commitment to medication adherence was influenced by the presence of family members with NCDs, as they had cared for them or witnessed the impact of NCD-related fatalities among relatives.

“I have taken care of my grandmother for the past 12 years. I had to carry her inside and outside of the room/house. Once she fainted and she could not move half of her body. Seeking that, I am motivated to keep up with my clinical visits and medication”

(HTN Patient, 66 years Female, HP)

“My husband fell very ill; I had to take care of him, help him go to the toilet and clean up, feed him. I went to Madhyapur Thimi for a check-up because I could not sleep well at night. This all led me to start medicine.”

(HTN Patient, 60 years, Female, HP)

Participants added that the personal experiences and their fear of developing complications or dying resulted in them becoming more conscious of managing their NCD conditions. Specifically, many participants expressed that fear of disease consequences led them to emphasize control measures like regular monitoring, treatment, follow-up, medical adherence, and lifestyle modification, resulting in increased utilization of NCD services.

“I take medicine with the fear of losing my life. The doctor informed me that arriving just one minute late could have cost me my life. I worry about not getting medicines from here; I wonder who will provide them. As a result, I am now almost disabled and unable to be economically active.”

(HTN Patient, 50 years, Female, HP)

#### Health Service Provider’s advice

Most participants reported receiving guidance from their health service providers regarding medication adherence, regular check-ups, adopting a healthy diet, and abstaining from alcohol and tobacco. Some participants indicated they were willing to pay for treatment when advised by their service providers. Experiencing health improvements as a result of following these recommendations positively impacted their utilization of services. A significant number of participants expressed a preference for revisiting health facilities when provided with dietary and lifestyle modification advice.

“The Health service providers advise avoiding oily, spicy foods and fatty meats. They helped me to be updated about my health. If my blood pressure reading is high, they suggest the dos and don’ts and encourage me to take medicine.”

(HTN Patient, 66 years, Female, HP).

“The medicines from the doctor have improved my health and also, counseling and advice on diets like healthy foods, adding white meat instead of red meats, eggs, and more fluids, and avoiding oily, spicy foods.. I like coming back and consulting.”

(HTN Patient, 75 years, Female, PHCCs)

#### Confidence in Health Service Providers and services

Few participants expressed confidence in the healthcare services provided at the health facility. Many participants expressed confidence in the healthcare services provided by the health post. They felt assured of receiving necessary medications and appreciated the establishment of health facility in their area. Participants believed that the health services and medicines available at the facility would help improve their health conditions and enable them to lead healthier lives.

“I feel that medicines and health services from here will help me to improve my health condition and live a healthy life.”

(HTN Patient, 50 years, Female, HP)

The accessibility of the health facility, particularly for those living nearby, reinforced their confidence and satisfaction with the services. They expressed satisfaction with the care they received, emphasizing the polite and supportive nature of the healthcare providers; along with advice on healthy diets and medications, contributed to their positive experiences. The proximity of the health facility also reduced the need for follow-up calls, as they could easily access services when needed. A Participant reported a strong trust in the health facility, noting that its government-run status further increased their confidence.

“I am confident on the services: first it is near to my place, second the health service providers attend us very well and ask about our concerns.. and then check our pressure. Instructions from him is doable and follow.”

(HTN patient, 40 years, Female, HP)

### Self-efficacy

#### Adherence to medication and treatment

Many participants indicated that they began taking medication for their condition promptly upon diagnosis and reported positive impacts on their health from the medication’s use.

“My pressure is better now. I feel that medicines and health services help me improve my health, and I’m confident that I will live a healthy life.”

(HTN Patient, 85 years, Male, HP)

#### Self-motivation

Participants reported testing devices to monitor their blood glucose levels. One reason that participants were motivated to adhere to treatment was their love for their family and their strong desire to live longer to witness the growth of their children’s offspring. To manage their health conditions, participants were also proactive and resourceful in the face of limited healthcare services. For example, One male participant with diabetes, described the absence of a laboratory service to assess blood sugar levels which he perceived to be a significant gap in the healthcare services provided by PHCCs. To manage this gap, he purchased his own blood sugar monitoring device.

“They do not have a lab service to assess blood sugar levels. Therefore, I have bought the device. Sometimes my daughter-in-law buys… I do the checking in every 15 days.”

(DM Patient, age missing, Male, PHCCs)

## DISCUSSION

Applying the Health Belief Model, this qualitative study identified multiple facilitators and barriers that patients face in utilizing NCD services at the primary health care level in Nepal. Participants often lacked knowledge and understanding of NCD services in their community. Participants who witnessed family members suffering from NCD complications due to delayed treatment and care described feelings of fear, underscoring the severity of the diseases. This fear prompted participants to prioritize disease management, leading to better self-management through regular monitoring and treatment adherence. The facilitators for NCD management at the health system level included access to free medicines through health insurance schemes, proximity to healthcare facilities, short waiting times, and positive patient-provider interactions. Supportive family and peers also played a vital role in NCD health care utilization. Additionally, certain cues to actions such as medicine monitoring by family members, healthcare provider’s advice, and self-efficacy motivate participants to prioritize treatment adherence. However, barriers such as inadequate health services, geographical inaccessibility, financial constraints, and few reminders and follow-ups from healthcare providers hindered NCD service utilization.

Participants who refrained from seeking healthcare often did not perceive hypertension as a serious medical condition(23). Similar patterns have been observed in studies conducted in Nigeria, the United States, Malaysia, and Nepal,(24–27) where asymptomatic individuals similarly perceived themselves as being in good health and not seeking care. The asymptomatic nature of the disease contributes to a delay in the initiation of healthcare-seeking behavior, with individuals prioritizing symptomatic treatment approaches (26, 25). In our study, participants had inadequate knowledge and misconceptions about NCDs treatment, which made them hesitant to lifetime commitment to medication. These findings are congruent with previous studies from Nepal(28, 29) and other LMICs (30–32). Individuals lacking literacy skills often encounter challenges in accessing relevant information and knowledge(33), impeding the timely initiation of disease treatment and control(34). Furthermore, people with less education tend to favor alternative treatment modalities(31, 28), a phenomenon observed not only in our study but also prevalent in other low- and middle-income countries (LMICs)(35–37).

In our study, fear of complications or death, experiences of illness within the family, past negative experiences or deaths, and the presence of other conditions (co-morbidity), were factors encouraging individuals to seek healthcare and treatment adherence. These findings align with similar studies conducted in Jamaica,(38) Singapore,(39) India(40), Nigeria(41, 42) and South Africa(41, 42) suggesting that perceived severity acts as a motivator for medication adherence. In our study, participants who received support from their family and peers had enhanced medication adherence. This aligns with findings from previous study in India(43) and other South Asian countries(32), where medication adherence, regular medicine refills, and hospital visits were positively associated with receiving family and peer support. Like our study findings, studies have also emphasized the role of family member support in utilization of hypertension medical care. To prevent NCDs, families and communities must be educated(36, 44).

The presence of a social health insurance scheme that comprehensively covered healthcare costs and the availability of medications served as motivating factors for individuals seeking NCD care at primary healthcare facilities. However, shortages of medicines force participants to make payments directly from their pockets and frequently depend on private healthcare facilities. Out-of-pocket expenditure continues to be the primary method of financing healthcare in Nepal(45, 46). medicines short in supply and irregularity of medicine supply shows lack of proper coordination with the health system regarding medicines purchase, delayed procurement, delayed budget allocation, and inadequacy of the budget(46). Therefore, an in-depth analysis of needs, improved coordination between the center and districts, increased procurement efficiency, and proper planning of shortages and expiring medicines is paramount. Receiving general health services and available medicines at the health facility encourage participants to visit health services, which cause overall satisfaction with the service provided at the health centers(47).

Proximity to healthcare facilities emerged as a critical factor in facilitating service utilization at the primary healthcare level in Nepal. In Nepal, where geographical barriers might exist due to hilly terrain, having health facilities nearby becomes crucial to reduce travel time, costs, and logistical challenges, making it easier for individuals to seek and receive care. This finding is unsurprising and aligns with what is often observed in the healthcare system worldwide(48–50). Similar patterns were observed in studies conducted in various other countries such as India, Pakistan and South Africa(30, 51, 52).

Numerous participants, from both PHCCs and health posts, have expressed that the minimal waiting time for consultations, health examinations, and medication dispensing has served as a motivating factor for them to persist in seeking assistance at the health facility. The findings resonate with a study in Iraq(53). Reduced waiting times for check-ups enhance service satisfaction, encouraging participants to visit healthcare facilities regularly; however, participants express significant concerns about inadequate communication, minimal to no counseling, and insufficient follow-up from health service providers. The behavior and conduct of healthcare providers significantly influence patient perceptions of health services. Establishing rapport and fostering trust with participants positively impact their perception of the quality of care(43). Similarly, in a study(54), participants indicated that they perceived the health workers as being responsible for and most knowledgeable about the patient’s health.The attitude of the service providers and inadequate communication during consultations are some of the constraints in accessing health care services for participants in rural South India(30).

In the present study, a shortage of continuous medicine supply was found to be a barrier to service utilization. Periodic shortages of medicine enforced medicine purchases from private pharmacies; thus, medication costs or affordability were seen as barriers to adherence among the participants, which is also found in another study in Nepal.(8) The cost of NCD treatment for the economically poor population is challenging, which caused non-adherence to service utilization(8).

Participants visited PHCCs to access various health services such as checkups, medications, laboratory tests, and social insurance benefits, which were exclusively offered here only. However, the NCD services provided were often basic screenings and medication in some instances, specific centers lacked screening programs necessary for diagnosing NCDs, as reported by a study in Nepal(55). Several barriers to utilizing these services were identified, including the absence of reminders for medication and follow-up visits, insufficient information provided by healthcare providers, and patient dissatisfaction with the services rendered. The utilization of NCD services in Nepal through the PEN approach remains limited(9, 56) due to inadequately equipped and capacitated healthcare infrastructure(9). The scenario reflects a broader trend seen in LMIC(57), where health disparities and out-of-pocket health expenditures are rising, and Nepal is no exception.

Participants expressed concerns about out-of-pocket expenses related to medicine costs and treatment. The financial burden tends to escalate with the number of co-existing health conditions, contributing to increased out-of-pocket expenditure with the number of co-morbidities(58). Similar observations were noted in a study conducted in India, where the cost of medication constituted the most substantial proportion of expenses for participants, followed by spending on healthcare providers(59). This situation disproportionately affects individuals from rural areas due to the additional cost of transportation to reach healthcare facilities. These findings collectively highlight the overwhelming reliance on out-of-pocket expenditures to finance healthcare, particularly impacting economically disadvantaged populations. The reliance exacerbates health inequities, creating significant challenges, particularly for financially vulnerable individuals, and contributes to the overall financial strain associated with accessing healthcare services(60).

In Nepal, there have only been a few studies conducted investigating the facilitators and barriers to NCD service utilizations from the patient perspective. We utilized an iterative process of data analysis through code saturation to document the range of perceptions of new issues. This study reflect the wide geography of Nepal as participants and providers from all seven provinces were interviewed. There are a few limitations to this study. First, the study’s results may not reflect the views of patients who did not utilize health care, as the participants were recruited from primary health care facilities. Second, participants included those with diabetes, hypertension, and COPD, but not cancer patients. Third, HBM simplifies healthcare decision-making by focusing primarily on individual perceptions, potentially overlooking broader socioeconomic and environmental factors that influence access to and utilization of healthcare. Lastly, the interviews were conducted at the health facilities, so participants may have been reluctant to report some of the health-worker related barriers due to social desirability bias.

## Conclusions

The study elucidates the barriers and facilitators to the utilization of NCD services at the primary healthcare level in Nepal, from the patient’s perspective. Barriers include inadequate infrastructure, geographical inaccessibility, financial constraints, and insufficient reminders from healthcare providers. However, factors influencing utilization include limited knowledge about healthcare, fear among those experiencing adverse outcomes due to delayed treatment in family members, and positive health system factors like free medicines, proximity, and supportive interactions. Family and peer support, along with family reminders and provider advice that encourages treatment adherence. To enhance NCD service utilization in Nepal, comprehensive health education is needed to raise awareness about availability of healthcare services, establish community outreach for remote areas, promote patient empowerment, and invest in healthcare infrastructure including strengthening, peer and family support and reminders, and foster multi-sectoral collaboration.

## Figures and Tables

**Table 1:** Characteristics of study participants (n = 35)

Characteristics	n (%)
**Age category**	
35 < 44	3 (8.5)
45 < 54	8 (22.8)
55 < 64	6 (17.1)
65 < 74	8 (22.8)
75 years or older	4 (11.4)
**Sex**	
Female	19 (54.3)
Male	16 (45.7)
**Education level**	
No formal education	16(45.7)
Basic Literacy	7(20)
Primary education	6(17.14)
Secondary education	4(11.4)
Higher education	2(5.7)
**Family structure**	
Nuclear family	10 (28.5)
Joint/extended family	25 (71.4)
**Type of NCDs**	
Hypertension	22 (62.9)
Diabetes Mellitus	6 (17.1)
Chronic Obstructive Pulmonary Diseases	2 (5.7)
Hypertension and Diabetes Mellitus	3 (8.6)
Hypertension and Chornic Obstructive Pulmonary Disease	2 (5.7)
**Family History of NCD**	
Hypertension	10 (28.5)
Diabetes Mellitus	4 (11.4)
Chronic Obstructive Pulmonary Diseases/ Asthma	3 (8.5)
None	15 (42.8)
**Age category**	
Others (arthritis, gastritis)	2 (5.7)
**Occupation**	
Agriculture	12(34.28)
Homemaker	13(37.14)
Foreign Employment (Migrant worker)	2(5.71)
Retired	1(2.85)
Bank	1(2.85)
None	6(17.14)

**Table 2:** Presents the themes, sub-themes identified and derived under the HBM constructs.

HBM Constructs	Themes	Sub-themes	Illustrative Quotes
**Perceived Barriers**	A. Barriers	• Inadequate Health services (Medications, equipments)	*“Medicine is not available all the times. They ask me to buy medicine; especially expensive ones from other place.”* *“Lab services like blood sugar tests for diabetes are not available in this health post, so we have to go to Beni Hospital. Also, medicines are not available and we have to travel a long distance for it.”*
		• Inaccessibility and Affordability	*“I wish the medicine and service was available here. The roads are not easy to travel nor do I have any vehicles. It takes an hour long to reach the pharmacy.”*
		• Low knowledge of NCD care	*“I am not aware of the NCD services offered through HF by Nepal Government. I hear about PEN program today only.”*
		• Inadequate health-related information from Health Service Providers	*“Health service providers have not given any information or advice on my disease or the health care I need. I have not received any information.”*
		• No reminders/followups	*“There has not been any contact from the Health Service Providers. We come ourselves for checkups and follow ups.. There’s no follow up service.”*
**Perceived Susceptibility**		• Limited knowledge about NCD care	*“I donot know anything about Hypertension.. I am not aware of the NCD services offered here.”*
**Perceived Severity**		• Witness adverse effects in family • Fear of complications, death, disability, Aggravated Symptoms	*“I take medicine with a fear of losing my life.”* *“I come for a checkup due to the fear of developing complications such as paralysis or losing mobility.”*
**Perceived Benefits**	B. Facilitators	• Accessibility	*“After the construction of the health post in our village, it has been very easy for us to access the health services.”*
		• Less waiting time	*“I get the services on time, I don’t have to wait, they respond immediately..”*
		• Services at low-cost/free services	*“We don’t have to pay for the check-ups. And also, we get medicine for free. But, if we go to the nursing home, we have to pay the doctor’s fees.”*
		• Positive interaction with Health Service Providers	*“Health service providers have good knowledge and I’m happy with the service provided by them.”*
		• National Health Insurance Scheme	*“Insurance has opened access to all kinds of (health) checkups, medicines, and other health-related services. The treatment cost is cheap, I tested my sugar for Rs.40 ($0.03).*
		• Support from family and peers	*“My daughter-in-law, sons, and grandchild continuously remind me to have timely health-check up and medication.”*
**Cues to Action**		• Experience with Illness	*“I was afraid that something bad would happen or I might be disabled so, I thought I had to take medicine and started taking medicine and visit the health center.”*
		• Health Service Providers’ advice	*“The health service providers have been advising on being active and regularly exercising or go for morning walks. I am reducing my consumption of alcohol and I’m trying to quit it. Thus, my health is improving well.”*
**Self-Efficacy**		• Confidence in Health Service Providers	*“I trust the health service offered by HW in this HF that I will be cured.”*
		• Adherence to medication	*“I was afraid that something bad would happen or I might be disabled so, I thought I had to take medicine and started taking medicine.”*
		• Self-motivation	*“For 3 years, I have been taking medicine regularly. I adhere to health service providers’ advice and come for regular follow-up visits.”*

## Abbreviations

AHW: Auxiliary Health Worker
ANM: Auxiliary Nurse Midwife
CVD: Cardiovascular Diseases
COPD: Chronic Obstructive Pulmonary Disease
DM: Diabetes Mellitus
DOHS: Department of Health Services
EDCD: Epidemiology and Disease Control Division
HP: Health Post
HA: Health Assistant
HTN: Hypertension
HSP: Health service providers
HBM: Health Belief Model
IDI: In-depth Interview
KII: Key Informant Interview
MoHP: Ministry of Health and Population
NCD: Non-communicable Diseases
NHRC: Nepal Health Research Council
PEN: Package of Essential non-communicable diseases
PHCCs: Primary Health Care Centers
RA: Research Assistant
UHC: Universal Health Coverage
WHO: World Health Organization
